# In vitro evaluation of the effect of galectins on *Schistosoma mansoni* motility

**DOI:** 10.1186/s13104-023-06530-9

**Published:** 2023-10-10

**Authors:** Tomoharu Takeuchi, Risa Nakamura, Megumi Hamasaki, Midori Oyama, Shinjiro Hamano, Tomomi Hatanaka

**Affiliations:** 1 Laboratory of Biochemistry, Faculty of Pharmacy and Pharmaceutical Sciences, Josai University, 1-1 Keyakidai, Sakado, Saitama 350-0295 Japan; 2 Department of Parasitology, Nagasaki University, Institute of Tropical Medicine (NEKKEN), 1-12-4 Sakamoto, Nagasaki, Nagasaki, 852-8523 Japan; 3 The Joint Usage/Research Center on Tropical Disease, Nagasaki University, Institute of Tropical Medicine (NEKKEN), 1-12-4 Sakamoto, Nagasaki, Nagasaki, 852-8523 Japan; 4 Leading Program, Graduate School of Biomedical Sciences, Nagasaki University, 1-12-4 Sakamoto, Nagasaki, Nagasaki, 852-8523 Japan; 5 School of Medicine, Tokai University, 143 Shimokasuya, Isehara, Kanagawa 259-1193 Japan

**Keywords:** Galectin, Glycan, Lectin, Parasite, *Schistosoma mansoni*

## Abstract

**Objective:**

Galectins are sugar-binding proteins that participate in many biological processes, such as immunity, by regulating host immune cells and their direct interaction with pathogens. They are involved in mediating infection by *Schistosoma mansoni*, a parasitic trematode that causes schistosomiasis. However, their direct effects on schistosomes have not been investigated.

**Results:**

We found that galectin-2 recognizes *S. mansoni* glycoconjugates and investigated whether galectin-1, 2, and 3 can directly affect *S. mansoni in vitro*. Adult *S. mansoni* were treated with recombinant galectin-1, 2, and 3 proteins or praziquantel, a positive control. Treatment with galectin-1, 2, and 3 had no significant effect on *S. mansoni* motility, and no other differences were observed under a stereoscopic microscope. Hence, galectin-1, 2, and 3 may have a little direct effect on *S. mansoni*. However, they might play a role in the infection in vivo via the modulation of the host immune response or secretory molecules from *S. mansoni*. To the best of our knowledge, this is the first study to investigate the direct effect of galectins on *S. mansoni* and helps in understanding the roles of galectins in *S. mansoni* infection in vivo.

**Supplementary Information:**

The online version contains supplementary material available at 10.1186/s13104-023-06530-9.

## Introduction

Schistosomiasis caused by *Schistosoma* spp., such as the parasitic trematode *Schistosoma mansoni* is a neglected tropical disease (NTD) associated with resource-poor regions [1]. *Schistosoma* spp. infection results in bowel, liver, spleen, or urogenital diseases, which could worsen poverty. Over 250 million people are infected with *Schistosoma* spp., and the WHO 2021–2030 road map for NTDs includes elimination of schistosomiasis [2]. Praziquantel (PZQ) is the primary drug for effectively controlling schistosomiasis and exhibits anti-parasitic activity via a transient receptor potential channel [3]. However, the possibility of developing drug resistance to PZQ cannot be ignored. Continuous research for the development of an alternative treatment and understanding the host defense mechanism against *Schistosoma* spp. is required [1]. Therefore, glycan-mediated host-parasite interactions have garnered interest in recent years [4].

Galectins are a family of sugar-binding proteins widely distributed among various species, and more than 10 galectin proteins are found in mammals. They bind to cell surface or extracellular glycoconjugates having β-galactoside structure(s), and function in various biological phenomena e.g. development, cancer, and immunity [5–7]. Galectins can affect a variety of host immune cells, such as T cells, B cells, NK cells, macrophages, and neutrophils and exert their immunomodulatory activities, e.g. human galectin-2 induces T cell apoptosis [8]. They directly bind to bacteria, viruses, and parasites and can affect their pathogenicity [9, 10]. Fungal galectin CGL2 binds to *Caenorhabditis elegans* cell surface glycoconjugates, inhibits their development and ultimately kills them [11]. Sheep galectin-11 directly binds to the parasitic nematode *Haemonchus contortus* and suppresses larval growth and development [12]. Mammalian galectin-2 directly binds to an invertebrate-specific Galactoseβ1-4Fucose disaccharide epitope and has demonstrated potential anti-parasitic activity in an experiment performed using *Caenorhabditis elegans* [13].

The trematode *S. mansoni* expresses Lewis X epitope (Galβ1–4(Fucα1–3)GlcNAc) and LacDiNAc epitope (GalNAcβ1-4GlcNAc) but not the Galβ1-4Fuc epitope [14]. However, given the sugar-binding specificity of galectin-2 [15], it is presumed that galectin-2 binds to *S. mansoni* glycoconjugates containing Lewis X or LacDiNAc epitopes. Hence, galectin-2 may directly affect this parasitic trematode. In addition, *S. mansoni* glycoconjugates are also recognized by galectin-3 and it binds to the worm surface; however, its direct effect on this parasite remains undetermined [16]. Galectin-1 does not recognize the *S. mansoni* glycoconjugates [16]. However, its expression is upregulated during *S. mansoni* infection, suggesting its association with the infection [17]. Therefore, it could be hypothesized that galectin-1, 2, and 3 play a role in *S. mansoni* infection. In this study, we investigated the binding ability of galectin-2 with *S. mansoni* glycoconjugates and the potential effects of galectin-1, 2, and 3 on *S. mansoni in vitro* with a focus on its motility.

## Materials and methods

### Preparation of recombinant galectin proteins

Mouse galectin-1C2S, galectin-2, and galectin-3 recombinant proteins were expressed in *Escherichia coli* and affinity-purified with an asialofetuin- or Galβ1-4Fuc-immobilized sepharose column basically as described previously [13]. After sterilization by filtration, each recombinant protein (1 µg) was subjected to SDS-PAGE and stained with Bio-Safe™ Coomassie (Bio-Rad, Hercules, CA, USA). The concentration of each purified protein was determined using a Bio-Rad Protein Assay (Bio-Rad) with bovine serum albumin (BSA) as the standard.

### Lectin blotting

Adult *S. mansoni* and mixed stage of *C. elegans* were prepared and lysed by sonication and boiling in SDS-PAGE sample buffer as described previously [13]. Worm extracts and BSA (50 ng) were separated by SDS-PAGE and then subjected to lectin blotting, as described previously [18]. HRP-labeled galectin-2 was prepared using a peroxidase labeling kit – NH_2_ (Dojindo, Kumamoto, Japan), according to the manufacturer’s instructions.

### Affinity chromatography

Adult *S. mansoni* parasites were suspended in phosphate-buffered saline (PBS; 8.1 mM Na_2_HPO_4_, 1.47 mM KH_2_PO_4_, 137 mM NaCl, and 2.68 mM KCl; pH 7.4) containing 1 mM EDTA (PBS-EDTA) and disrupted by sonication. The parasite extract was subjected to affinity chromatography using a galectin-2-immobilized column, as described previously [13]. In brief, the parasite extract was applied to an immobilized galectin-2 column (bed volume 1 mL; 15.8 mg protein/mL gel). After washing the column with PBS-EDTA, the adsorbed materials were specifically eluted with PBS-EDTA containing 0.1 M lactose. The collected fractions were subjected to Coomassie Brilliant Blue (CBB) staining and Pro-Q Emerald staining using the Pro-Q Emerald 300 Glycoprotein Gel Stain Kit (Thermo Fisher, Waltham, MA, USA) according to the manufacturer’s instructions.

### Identification of galectin-2-binding *S. mansoni* protein

**Fig. 1:**
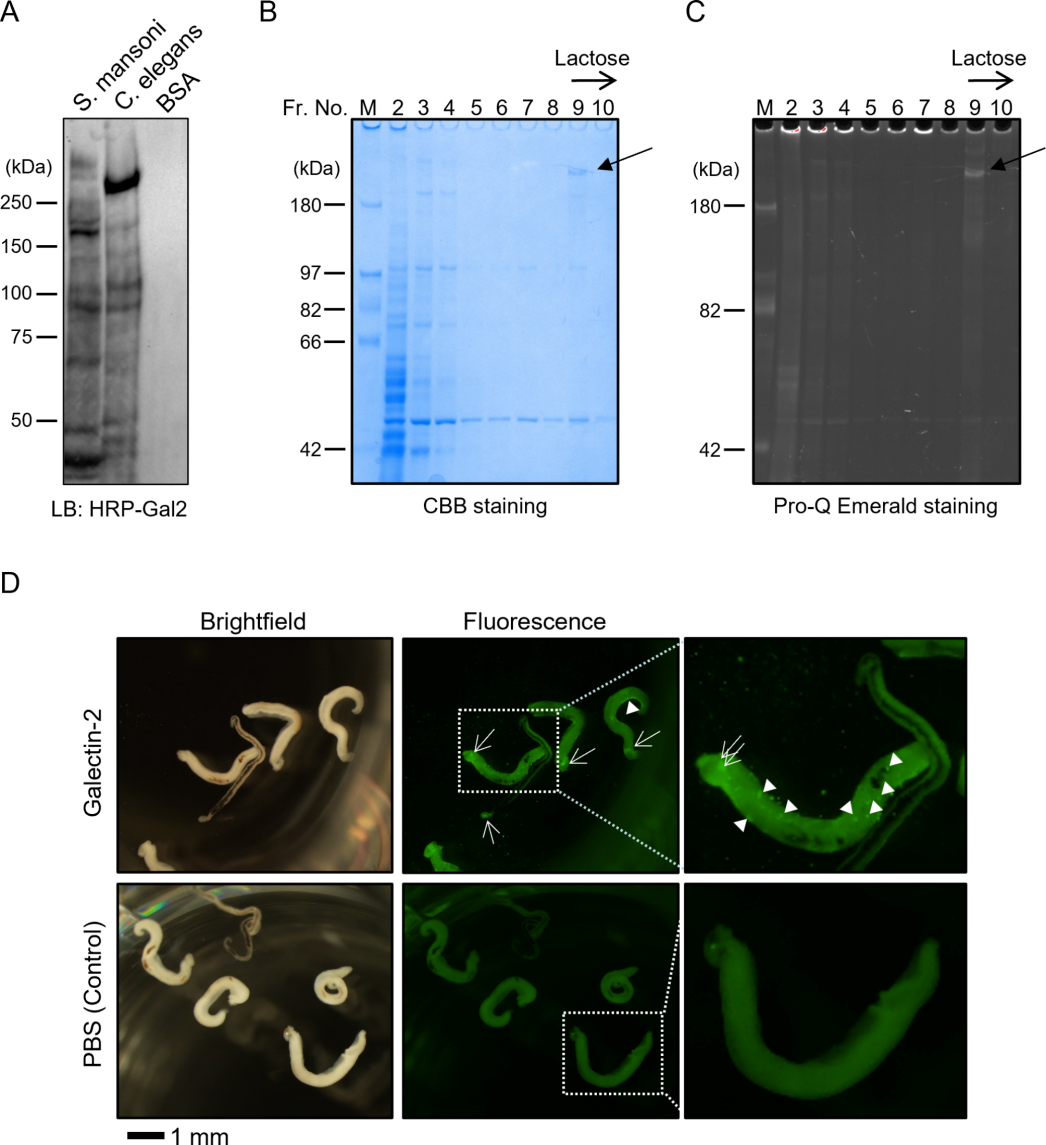
Investigation of the binding ability of galectin-2 to *S. mansoni* glycoconjugates. **(A)***S. mansoni* extracts were subjected to SDS-PAGE and lectin blotting using HRP-labelled galectin-2. **(B, C)***S. mansoni* protein bound by galectin-2 was isolated by affinity chromatography using an immobilized galectin-2 column and eluted with lactose. It was subjected to SDS-PAGE and **(A)** CBB-staining or **(B)** Pro-Q emerald staining. The arrows in B and C indicate galectin-2-binding glycoprotein identified by mass spectrometry. **(D)** Adult pairs of *S. mansoni* were stained with Alexa488-labeled galectin-2 or treated with PBS (negative control). Arrows and arrowheads indicate galectin-2-dependent staining of the worms

The portion of the gel indicated in Fig. 1 (B) was excised, and the proteins therein were identified by nanoLC–MS/MS via a contract analytical service from Japan Proteomics (Miyagi, Japan).

### Lectin staining

Alexa488-labeled recombinant mouse galectin-2 protein was prepared as described previously [13]. Adult pairs of *S. mansoni* were transferred into each well of a 24-well plate containing 450 µL of RPMI-1640 medium (Fujifilm Wako, Tokyo, Japan) with antibiotics (1× penicillin/streptomycin purchased from Fujifilm Wako). Next, 50 µL of 1 mg/mL Alexa-labeled galectin-2 protein dissolved in PBS was added to the medium. After incubation at room temperature for 2 h, the worm was washed two times with Dulbecco’s PBS (D-PBS) and images were captured using an Olympus SZX16 stereo microscope (Olympus, Tokyo, Japan) equipped with a GFP filter unit and DP74 digital camera. The cellSens software (Olympus) was utilized for image acquisition.

### Hemagglutination assay

The hemagglutination assay was performed, as basically described previously [19]. In brief, rabbit erythrocytes treated with trypsin and glutaraldehyde were mixed with each galectin proteins in 100 µL of PBS containing 50 µg/mL of BSA in a 96-well V-shaped titer plate, and then incubated at room temperature for 1 h.

### Maintenance and preparation of *S. mansoni* parasites

The Puerto Rican strain of *S. mansoni* was maintained in the animal facilities of Nagasaki University by passage through *Biomphalaria glabrata* snails and ICR mice. To prepare *S. mansoni*, the mice were percutaneously infected with 250 cercariae. Approximately eight weeks after infection, adult worms were obtained by portal vein perfusion [20].

### Motility assay of *S. mansoni* parasites

An adult pair of *S. mansoni* was transferred into each well of a 24-well plate containing 450 or 495 µL of RPMI-1640 medium (Fujifilm Wako) without serum but with antibiotics and antimycotics (1× penicillin/streptomycin/amphotericin B purchased from Nacalai (Kyoto, Japan)). Next, 50 µL of 100 µM each galectin protein dissolved in PBS was added to the medium to achieve a final concentration of 10 µM. As the positive control, 5 µL of 1 or 10 mM PZQ (Sigma-Aldrich, St. Louis, MO, USA) or dimethyl sulfoxide (DMSO) was added to the medium, resulting in a total volume of 500 µL and a final PZQ concentration of 10 or 100 µM. Since PZQ is insoluble in water, it was dissolved in dimethyl sulfoxide (DMSO). After incubation at 37 °C under an atmosphere containing 5% CO_2_ for 24 h, the motility of adult pairs of *S. mansoni* was examined (N = 3 for PZQ, N = 4 for each galectin) using an Olympus SZ61 stereo microscope (Olympus) equipped with a Swiftcam SC1603 digital camera (Swift, San Antonio, TX, USA), and pictures and videos were captured using the Swift imaging software (version 3.0). Motility was scored on a 5-point scale (4: normal motility; 3: reduced motility; 2: uncoordinated motility; 1: severe reduction of motility; 0: a complete absence of motility), as previously described [21].

### Statistics

Microsoft Excel was used for statistical analysis. Data are expressed as mean ± S.D. and were analyzed using Student’s t-test. *p*-values < 0.05 were considered statistically significant.

## Results

### Investigation of the binding ability of galectin-2 to *S. mansoni* glycoconjugates

We first investigated whether *S. mansoni* glycoconjugates could be recognized by galectin-2. Lectin blotting using HRP-labeled galectin-2 showed that not only the proteins in the *C. elegans* extract but also those in the *S. mansoni* extract were associated with positive signals (Fig. 1A). No such signal was observed in BSA, a negative control without glycosylation, suggesting that galectin-2 bonded with *S. mansoni* glycoconjugates. To clarify, *S. mansoni* proteins bound by galectin-2 were isolated by affinity chromatography and subjected to SDS-PAGE followed by CBB-staining or Pro-Q Emerald staining (Fig. 1B and C, respectively). The protein (> 200 kDa) was bound by galectin-2 in a β-galactoside-dependent manner since it was eluted by the addition of lactose. It was also stained with the glycoprotein staining reagent Pro-Q Emerald and was identified by nanoLC–MS/MS as alpha-2-macroglobulin (Smp_089670 in WormBase Parasite; https://parasite.wormbase.org/index.html) [22]. Lectin staining using fluorescently labeled galectin-2 showed that it could bind to the worm (Fig. 1D). We observed background fluorescence that was detectable in fluorescently labeled galectin-2-treated worms, as well as in PBS (control)-treated worms. However, we also observed galectin-2-dependent signals, particularly around the pharynx of male and female worms (indicated by arrows in Fig. 1D). Additionally, we noticed scattered fluorescent signals (indicated by arrowheads in Fig. 1D) in galectin-2-treated worms, which may be attributed to the endocytosis of galectin-2 by worm cells. Therefore, galectin-2 may bind to *S. mansoni* glycoconjugate(s) and directly influence the infectivity of this parasitic trematode.

### Evaluations of purity and biological activity of prepared galectin proteins

**Fig. 2:**
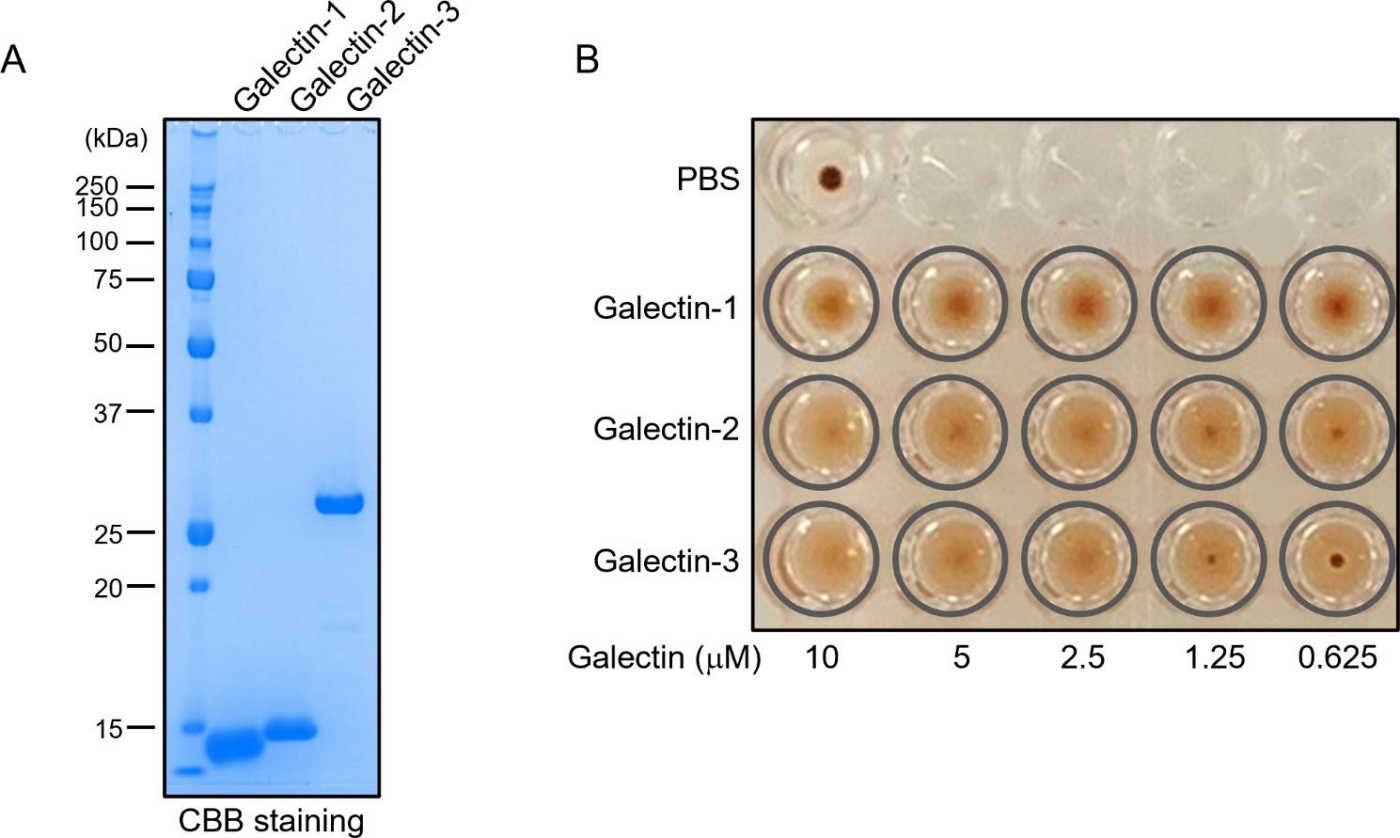
Evaluations of purity and biological activity of prepared galectin proteins. **(A)** Recombinant galectin proteins were subjected to SDS-PAGE and CBB-staining. The theoretical molecular weight of each recombinant galectin protein is as follows: galectin-1, 14.8 kDa; galectin-2, 14.9 kDa; galectin-3, 27.4 kDa. **(B)** Indicated final concentrations of galectin proteins were mixed with rabbit erythrocytes and incubated for one hour. Images were captured using a digital camera. The wells corresponding to agglutination are indicated by grey circles

To investigate the effect of galectin-2, galectin-1, and 3 on *S. mansoni in vitro*, we prepared pure and active recombinant galectin proteins. Protein purity was confirmed by SDS-PAGE and CBB staining, and a single band was observed for each protein (Fig. 2A). Their sugar-binding activities were confirmed by a hemagglutination assay, and the prepared proteins were effectively agglutinated at the concentration used for the motility assay or lower values (Fig. 2B).

### Effects of galectin proteins on the relative motility of adult *S. mansoni*

**Fig. 3:**
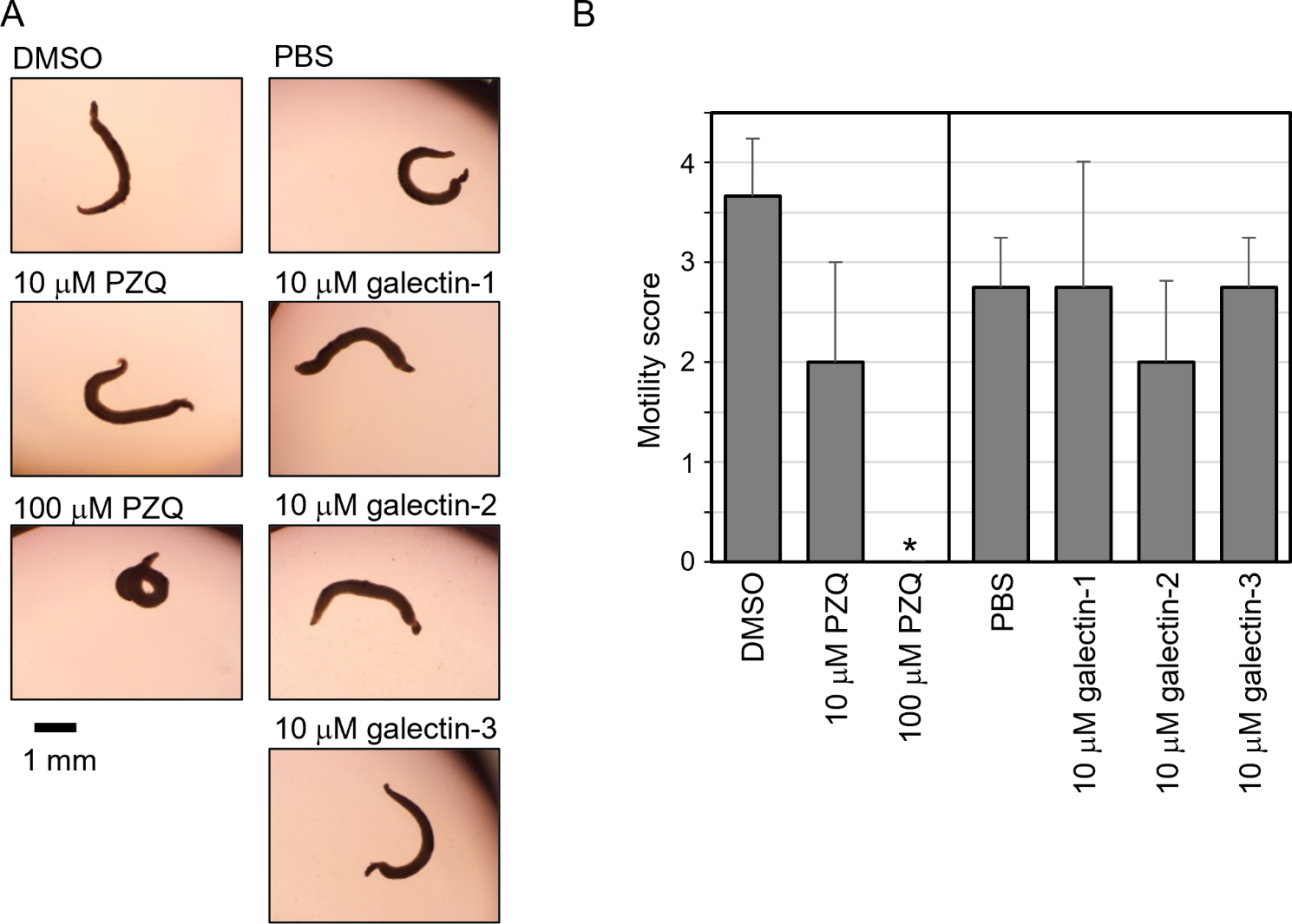
Effects of galectin proteins on the relative motility of adult *S. mansoni*. Adult pairs of *S. mansoni* (n = 3 or 4) were treated with praziquantel (PZQ; positive control) or galectin proteins for 24 h at 37 °C, 5% CO_2_. Conditions **(A)** and motility **(B)** of worms were examined under a stereomicroscope. Data are expressed as the mean ± S.D. *p < 0.05, vs. DMSO

Adult pairs of *S. mansoni* were treated with the prepared galectin proteins or PZQ, which is known to suppress worm motility [3] as a positive control. *S. mansoni* parasites treated with each galectin were observed to be normal, whereas those treated with high concentrations of PZQ (100 µM) were abnormally rounded (Fig. 3A; and Supp. videos 1–7). Parasite motility was then assessed (Fig. 3B). The treatment with each galectin was not significantly different from that of the negative control PBS, and no other differences were observed under a stereoscopic microscope. Meanwhile, PZQ treatment clearly suppressed motility and showed a significant difference compared to the negative control DMSO, indicating that the motility assay was conducted well. These results suggest that galectin-1, 2, and 3 did not prevent the motility of *S. mansoni* adult worms in vitro.

## Discussion

Since galectins can affect host immune cells and pathogens during the infection process, and such two-faceted functions of galectins can render data interpretation difficult in vivo, we prepared pure and biologically active recombinant galectin proteins and investigated the effect of galectin-1, 2, and 3 on the motility of *S. mansoni* for the first time to assess their direct effect; we found no direct effect of galectins on the worm. We also conducted the cytotoxicity assay by measuring lactate dehydrogenase (LDH) activity and found that galectins have no significant cytotoxic effect on the worm (data not shown). For the in vitro motility assay, 10 µM of each recombinant protein was used. The concentrations of galectin-1, 2, and 3 in human serum are as high as approximately 10 µg/mL, 1 µg/mL, and 1 µg/mL, respectively [23], which correspond to approximately 1 µM. The concentration of galectin-1, which is abundantly expressed in tissues, is ~ 40 mg/kg [24], which corresponds to approximately ~ 3 µM if we assume 1 kg of tissue to be 1 L. Therefore, the galectin concentrations used in this study were sufficiently higher than those in vivo, and the lack of significant effects on *S. mansoni* in the motility assay suggests that galectin-1, 2, and 3 mediate a little direct effect on this parasite in vivo.

The motility assay is useful as a simple system to examine the effects of compounds on *S. mansoni* [21]. However, it is possible that galectins may have other effects on the parasite in vitro if examined in more detail. Wang et al. reported a sophisticated *in vitro S. mansoni* culture system that could allow investigation of the egg production [25]. Using such an experimental system or examination of effects at the molecular level, for instance, those on genes and proteins, might help elucidate the direct effect of galectins on *S. mansoni in vitro* in the future.

Galectin-3 mediates *Schistosoma*-induced liver fibrosis via the regulation of host immune cells, especially macrophages [26]. Galectin-1 may also mediate *S. mansoni* infection via the regulation of host immune cells similarly to galectin-3. Galectin-2 has a direct inhibitory effect on parasites exerted via binding to the invertebrate-specific Galβ1-4Fuc glyco-epitope [13]. The trematode *S. mansoni* glycoconjugates do not express such glyco-epitopes [13, 14] but are recognizable by galectin-2. Although we observed no significant effect of galectin-2 on *S. mansoni*, it is possible that galectin-2 directly affects other parasites, particularly parasitic nematodes.

We found that galectin-2 could bind to multiple *S. mansoni* glycoconjugates and successfully identified one of its potential ligands, alpha-2-macroglobulin. Alpha-2-macroglobulin, which is described as a putative macroglobulin/complement in the WormBase Parasite, is a potential binding partner of galectin-2. Its localization is presumed to be in the cell surface and extracellular region in the database and is found in the vomitus of *S. mansoni* [27]. The precise function of *S. mansoni* alpha-2-macroglobulin has not yet been reported. This protein has an alpha-2-macroglobulin family domain according to SMART (http://smart.embl.de/) [28]. Since mammalian alpha-2-macroglobulin is a multifunctional protein with important roles in inflammation, immunity, and infection [29], secreted *S. mansoni* alpha-2-macroglobulin may affect the host and play a role in the infection process. Therefore, host galectin-2 may be involved in *S. mansoni* infection in vivo through its binding to alpha-2-macroglobulin. Such a study might reveal the role of galectin-2 in *S. mansoni* infection.

In conclusion, galectin-1, 2, and 3 had no particular effect on adult *S. mansoni* motility in vitro, although galectin-2 is found to recognize multiple *S. mansoni* glycoconjugates including alpha-2-macroglobulin in this study. The data presented here may help understand the functions of galectins in schistosomiasis. Further analysis of the role of these galectins in controlling *S. mansoni* infection via the modulation of host immune response or secretory molecules from *S. mansoni* will deepen our understanding of the host defense mechanisms against pathogens and, in the future, will aid in the control of pathogens.

### Limitations

In this study, we investigated the effect of galectin-1, 2, and 3 on adult *S. mansoni* motility in vitro. The possibility that they have some direct or indirect effects on *S. mansoni in vivo* cannot be ruled out.

### Electronic supplementary material

Below is the link to the electronic supplementary material.

Supplementary Material 1

Supplementary Material 2

Supplementary Material 3

Supplementary Material 4

Supplementary Material 5

Supplementary Material 6

Supplementary Material 7

Supplementary Material 8

## Data Availability

All data generated or analyzed during this study are included in this published article and its supplemental files.

## Abbreviations

BSA: Bovine serum albumin
CBB: Coomassie Brilliant Blue
DMSO: Dimethyl sulfoxide
NTD: Neglected tropical disease
PBS: Phosphate-buffered saline
PZQ: Praziquantel
