# Supplementary material for: In vitro evaluation of the effect of galectins on Schistosoma mansoni motility
Source: BMC Res Notes. 2023 Oct 10;16:266. doi: 10.1186/s13104-023-06530-9 (PMC10566010; doi:10.1186/s13104-023-06530-9)
Supplement: Supplementary file 8 — Supplementary Material 8 [file 13104_2023_6530_MOESM8_ESM.pdf]

Additional file

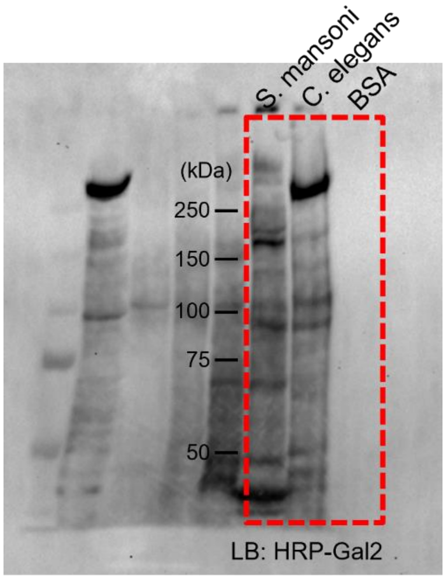

Fig. S1: Original blot image of figure 1A. The area shown in the figure 1A is indicated by the red dashed line.

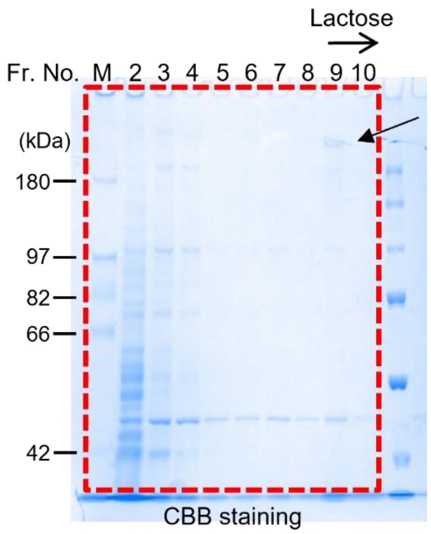

Fig. S2: Original gel image of figure 1B. The area shown in the figure 1B is indicated by the red dashed line.

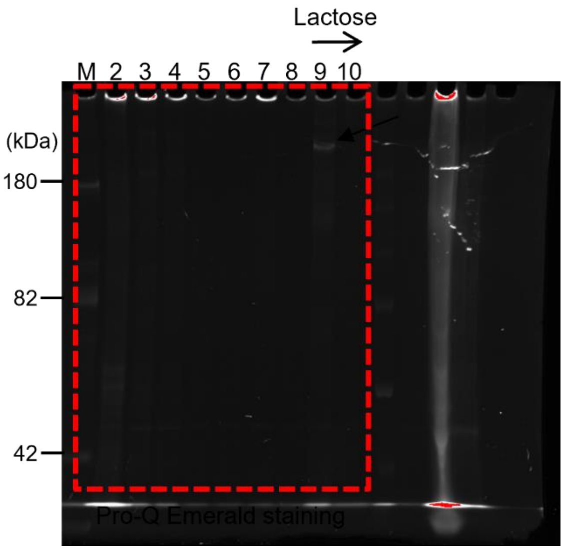

Fig. S3: Original gel image of figure 1B. The area shown in the figure 1C is indicated by the red dashed line.

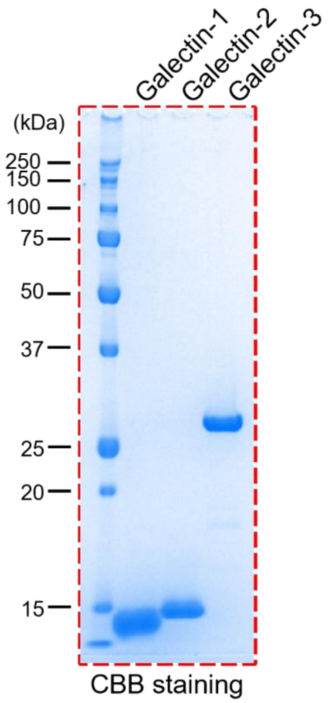

Fig. S4: Original gel image of figure 2A. The area shown in the figure 2A is indicated by the red dashed line.
